# Covid-19, not your normal flu: A case report on Covid-19 psychosis and mania in a Malaysian hospital

**DOI:** 10.51866/cr1369

**Published:** 2022-08-02

**Authors:** Rebecca Pei Ying Wong, Philip George

**Affiliations:** 1 International Medical University (IMU) Jalan Rasah, Bukit Rasah, Seremban, Negeri Sembilan, Malaysia. Email: WONG.PEIYINGREBECCA@student.imu.edu.my; 2 MBBS(Mang), MMed(Psych), Department Of Psychiatry, International Medical University, Jalan Rasah, Bukit Rasah, Seremban, Negeri Sembilan, Malaysia.

**Keywords:** COVID-19, Psychosis, Mania

## Abstract

Evidence suggests that acute severe coronavirus disease 2019 (COVID-19) may be associated with neuropsychiatric symptoms. This is a case report of a patient who had recently been infected with COVID-19 and had no history of psychiatric disorders presenting a few days after inpatient discharge from COVID-19 treatment with acute onset of psychosis and manic symptoms. This case illustrates the psychiatric presentation, possible causes, and management of post-COVID-19 psychosis.

## Introduction

Coronavirus disease 2019 (COVID-19) is an acute respiratory infection caused by severe acute respiratory syndrome coronavirus 2 (SARS-CoV-2).^1^ It was the isolated cause of a pneumonia outbreak in Hubei Province, China, in December 2019 and spread globally. To date, COVID-19 is responsible for over 162 million confirmed cases and 3.3 million deaths worldwide.^2,3^ SARS-CoV-2 is part of the orthocoronavirus subfamily and is the seventh coronavirus known to infect humans.^4^

Multiple presentations of COVID-19 have been identified, from flu-like presentations to neurovascular symptoms and psychiatric manifestations.^5^ We report on a case of post-COVID-19 psychosis and mania in a patient admitted to a local State Hospital in Malaysia.

## Case Presentation

Mr. K, a 54-year-old married, employed man was admitted from January 6, 2021, to January 29, 2021, for COVID-19 infection presenting with intermittent dizziness, a ‘floating feeling’, dyspnoea, chest tightness, dry cough, anosmia, generalised headaches, and myalgia. He was confirmed positive for COVID-19 infection via reverse transcription-polymerase chain reaction (PCR) test and required assisted ventilation (nasal prongs, high-flow performance devices) and high-dose dexamethasone tablets.

**Table 1:** Summary of Mr. K’s COVID-19 dexamethasone therapy.

Date range	Dexamethasone dose (mg)
6/1/2021-9/1/2021	16
9/1/2021-16/1/2021	24
17/1/2021-20/1/2021	20
21/1/2021-23/1/2021	16
24/1/2021-26/1/2021	12
27/1/2021-31/1/2021	8
1/2/2021-5/2/2021	4
6/2/2021-16/2/2021	8
17/2/2021-23/2/2021	4

His dexamethasone dose was tapered over the period of admission and was completed at home upon discharge. Mr. K was subsequently brought back to the hospital by his wife on March 2, 2021, with collateral history of gradual onset of abnormal manic-like, aggressive behaviour, such as throwing objects, disrupted sleep patterns over the previous 10 days, and psychosis for 3 days prior to admission.

Mr. K believed that God had sent him on a mission with the author of this case report and another friend to fight paranormal beings. He unrelentingly spoke on a series of topics including paranormal activities, spiritual strength, charities for blessings, and God. Other psychotic manifestations included persecutory delusions that ghosts and traditional healers wished to kill him and that spirits had been directing the hospital he attended for many years. He also claimed to see spirits in the hospital who took the form of dying people. He had second person auditory hallucinations described as terrifying and scary and refused to elaborate on them. There were no fluctuations of consciousness, and Mr. K was attentive throughout the interview. There were no symptoms or signs to suggest pre-existing dementia or delirium.

He had no prior psychiatric history or family history. Mr. K had a past medical history of hypertension, dyslipidaemia, and gout, and was on T. Aspirin 100 mg OM, T. amlodipine 5 mg OD, T. telmisartan 80 mg OD, T. allopurinol 300 mg OD, T. gemfibrozil 300 mg BD, and other unspecified supplements.

His mental state examination revealed a well-groomed, verbose, middle-aged man. He was quick to establish rapport and displayed overfamiliarity. He was restless, easily distracted, and uncooperative at times. He exhibited pressure of speech, flight of ideas, clang associations, neologisms, and tangential and circumstantial speech. As examples of neologisms, he claimed to have businesses called ‘project band' and 'kontelektor'. He laughed inappropriately; however, his mood was labile and congruent with his affect. He had no suicidal thoughts or plans. He had poor short-term memory, but his remote memory was intact. He was oriented to time, place, and person, with good judgement but poor insight. On physical examination, his lungs had reduced air entry bilaterally. ECG demonstrated T1 inversion in lead III. Lumbar puncture was normal and negative for viral meningitis. His chest radiograph showed left lower zone haziness, and a brain MRI with contrast showed bifrontal lobe lacunar infarcts. He was treated with an intravenous antibiotic, antiviral, oral sedative, and low-dose antipsychotics and made a good recovery.

**Table 2:** Patient’s blood panel, including complete blood count, electrolytes, D-dimer, C-reactive protein, serum cortisol, and liver and renal function tests.

Parameters	2/3/21	5/3/21	8/3/21	Normal range
White blood cell, ×l0^3^/uL	15.7(H)	10.4 (H)	9.9	4.0-10.0
Haemoglobin, g/dL	11.1 (L)	10.6 (L)	10.4 (L)	13.0-17.1
Platelets, ×l0^3^/uL	312	492	200	150-410
C-reactive protein, mg/L	158.3(H)	39.7 (H)	10.1 (H)	<3.0
D-dimer, ug/mL		0.9 (H)		<0.5
Serum cortisol (morning), nmol/L		760.4		140-690
Sodium, mmol/L	141	136	139	135-152
Potassium, mmol/L	3.7	3.5	3.7	3-5–5-4
Urea, mmol/L	5.8	4.9	4.5	1.7-6.4
Creatinine, mmol/L	101	68	69	40-170
Calcium, mmol/L	2.42			2.00-2.50
Magnesium, mmol/L	0.98			0.10-3.40
Phosphorous, mmol/L	1.09			0.50-3.00
Total protein, g/L	75			57-82
Albumin, g/L	47			32-48
Globulin, g/L	28			25-44
Bilirubin, umol/L	12			5-21
A/G ratio	1.7			0.9-1.8
Alkaline phosphatase, IU/L	106			46-116
Alanine aminotransferase, U/L	22			10-49

Legend: H: high; L: low

A urine toxicology panel was not requested. The patient’s leucocytosis suggested an infection or an underlying post-COVID-19 hyperinflammatory state, while his low haemoglobin was suspected to be due to his loss of appetite during COVID-19 infection. His D-dimer and C-reactive protein levels were elevated, which is not uncommon in patients infected with COVID-19, as these are nonspecific markers of inflammation. Depression and mania are common psychiatric symptoms seen in patients treated with corticosteroids. Therefore, morning serum cortisol was obtained and was elevated, which most likely suggested an iatrogenic cause of hypercortisolism as he had been prescribed high doses of steroids for his COVID-19 infection. His liver function tests, renal function tests, and electrolytes were not deranged. A chest radiograph taken on 3/3/21 revealed bilateral ground-glass opacities (GGOs), which suggested a widespread inflammatory or infiltrative lung disorder secondary to COVID-19 infection and ventilator-induced lung injury despite adequate saturation on room air. A computed tomography pulmonary angiogram revealed no pulmonary embolism. These pulmonary changes and blood results represented emerging evidence of an overexuberant inflammatory response that is seen in patients with severe COVID-19 infection.

The patient was treated at the emergency department with IV midazolam 5 mg OD PRN for his aggression. He was then admitted as an inpatient under liaison psychiatry for continued care and given T lorazepam 1 mg stat, IM haloperidol 2.5 mg stat, T risperidone 0.5 mg ON for his psychosis and abnormal behaviour, IV ceftriaxone 2g OD, IV acyclovir 500 mg TDS for any underlying occult infection, and T Aspirin 150 mg OD and T allopurinol 300 mg OD to control his comorbidities throughout his admission. He improved gradually over the following 7 days, and the antipsychotic medications were tapered off. At discharge, Mr. K was no longer aggressive but continued to show residual manic-like behaviour and was prescribed T aripiprazole 10 mg ON and T lorazepam 1 mg PRN.

**Table 3:** Progression of the patient’s behaviour in the ward.

Date	Observation
2/3/21	• No abnormal aggressive behaviour but manic-like behaviour present. • Slept well.
3/3/21	• The patient stood at window and recited the Quran. • He felt and appeared restless. • He tried to harm his wife. • Walked around ward with no intention. • IM haloperidol 2.5 mg was given, and the patient’s behaviour settled.
4/3/21	• Patient went to the toilet with wife to bathe, but he took the hot water and attempted to pour it on himself. • After calming the patient, he was able to sleep.
5/3/21	No abnormal aggressive behaviour, but residual manic-like behaviour present.
6/3/21	No abnormal aggressive behaviour, but residual manic-like behaviour present.
7/3/21	No abnormal aggressive behaviour, but residual manic-like behaviour present.
8/3/21	No abnormal aggressive behaviour, but residual manic-like behaviour present.

**Figure 1:**
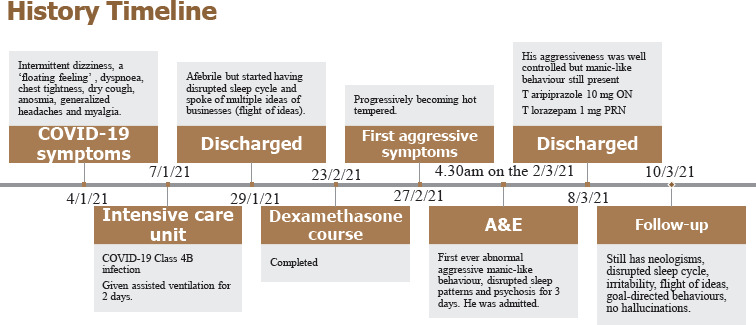
Timeline from the patient’s first COVID-19 symptoms to his first liaison psychiatry follow-up.

## Discussion

The 2019 novel coronavirus, once thought to be a ‘normal flu’, proved to be more, with evidence of neurological, cognitive, and psychological effects, even in patients who did not develop severe lung, heart, or circulatory problems.^6^

Our patient was admitted for COVID-19 infection treatment and was well upon discharge but returned with acute onset of psychiatric symptoms, including mania, delusions, and hallucinations. All investigations suggested no medical cause, and delirium was excluded. He was started on antipsychotics at low doses and recovered rapidly.

In June 2020, Lancet Psychiatry released a study on neurological and psychiatric complications in 153 people who were hospitalised with COVID-19 in the U.K. It reported that 39 people had altered mental status, 10 of whom had new-onset psychosis.^7^ Altered mental status is not uncommon in patients needing intensive care, but it predominates in the elderly, who often already have multiple medical comorbidities and polypharmacy. The authors observed a disproportionate number of neuropsychiatric signs in the young and cerebrovascular complications in the old. This might reflect the vulnerability of the central nervous system to COVID-19 infection or the increased accessibility of psychiatric aid to younger patients, whereas the elderly are often assumed to have delirium.^7^ A publication in Neuroscience Letters that reviewed 42 cases of psychosis reported in COVID-19-infected patients suggested that patients could exhibit a range of neuropsychiatric symptoms. However, the underlying pathological mechanisms have not yet been fully established.^8^

Much of the literature remains conceptual and conclusions are extrapolated from small studies conducted in the current pandemic and studies from previous epidemic viral infections.^7^ More research is needed to identify which pathogenic biomechanisms are driving neuropsychiatric associations. For now, this association is thought to be due to: 1) direct neuronal viral infection; 2) post-infectious neuronal autoimmunity; 3) vasculopathies, including those resulting from impaired coagulation; and 4) systemic (e.g., inflammatory) effects of a pervasive, severe pathogen and/or critical illness.^7,9^ Since 2010, research has focussed on the influence of inflammation and immunity in the onset of schizophrenia; therefore, a renewing interest in the idea that viruses can cause 'insanity' or, more specifically, psychoses, is not unusual.^10^ Meanwhile, a study on new-onset psychosis in people with COVID-19 in Spain reported that the time needed for an episode to develop, the length of the episode, and rapid recovery on low-dose antipsychotics helped to distinguish primary psychotic cases from secondary psychosis.^11^

Although steroids have been reported to trigger psychoses, the pathophysiology remains unclear. It is hypothesised that the synthetic steroids disrupt the cortisol pathway of the hypothalamic-pituitary-adrenal axis, resulting in mood disorders. Therefore, they create an imbalance between glucocorticoid and mineralocorticoid receptor stimulation, leading to cognitive impairment and emotional disturbances.^12^

A hypothesised pathophysiology of COVID-19 precipitating a manic episode is the hyperinflammatory cascade activating the kynurenine pathway and neurotropism, which causes a range of psychiatric presentations, including psychosis, bipolar disorder, depression, and suicide.^13,14^ A recent study discovered that inflammatory changes coincide with acute episodes of mania in patients with bipolar disorder.^15^

A lacunar infarct occurs due to an occlusion of a single penetrating artery and is responsible for one-quarter of cerebral infarctions. Interestingly, silent lacunar infarcts are more common because of their small size; they are often asymptomatic and only discovered on imaging as incidental findings. Depending on the area of brain involvement and the number of lacunar infarctions, they can lead to significant disabilities. However, cortical findings, such as behavioural changes, are absent as the infarction occurs only in subcortical areas of the brain.^16,17^ Therefore, in our patient’s case, his lacunar infarct may not have significantly contributed to his abnormal behaviour as this type of infarct is not associated with psychotic and manic-like symptoms. Moreover, his condition settled more rapidly than post-stroke psychosis, which would likely have had a longer duration of manifestation.

## Conclusion

COVID-19 has a significant impact on patients who are infected, even after physical recovery. As evidenced in other viral infections, COVID-19 can trigger psychiatric conditions, including psychoses and mania. Follow-up of patients in recovery after COVID-19 infection should include screening for psychiatric sequelae and appropriate investigations to rule out other causes, such as delirium. Early identification and treatment of this condition are likely to produce more favourable outcomes.
